# Determination of antioxidant activity and phenolic compounds for basic standardization of Turkish propolis

**DOI:** 10.1186/s13765-021-00608-3

**Published:** 2021-04-16

**Authors:** Aslı Özkök, Merve Keskin, Aslı Elif Tanuğur Samancı, Elif Yorulmaz Önder, Çiğdem Takma

**Affiliations:** 1 Bee and Bee Products Application and Research Center (HARUM), Hacettepe University, Ankara, Turkey; 2 Vocational School of Health Services, Bilecik Şeyh Edebali University, Bilecik, Turkey; 3 SBS Bilimsel Bio Çözümler Inc. Bee&You Propolis R&D Center, 34775, İstanbul, Turkey; 4 Department of Animal Science, Faculty of Agriculture, Ege University, İzmir, Turkey

**Keywords:** CUPRAC antioxidant capacity, HPLC, Propolis, Total flavonoid, Total phenolic

## Abstract

This study aimed to determine the standard amount of antioxidant content and compounds of the propolis for the standardization of propolis. For this purpose, the total flavonoids, total phenolic, CUPRAC antioxidant capacity content and the diversity of phenolic and flavonoid components of these propolis samples were found by HPLC determined at the 23 propolis samples which were collected different regions of Turkey. Beside that, the similarities and differences of these 23 provinces to each other according to their antioxidant capacities were investigated by multidimensional scaling analysis. The total flavonoid content in the propolis samples were determined between 21.28 and 152.56 mg CE/g. The total phenolic content in the propolis samples was found between 34.53 mg and 259.4 mg GAE/g. CUPRAC antioxidant capacity of the propolis samples and antioxidant range was found from 95.35 to 710.43 mg TE/g. Also, 4 flavonoid [Quercetin (min.1.12–max.4.14 mg/g), Galangin (min.0.72–max.40.79 mg/g), Apigenin (min.1.07–max.17.35 mg/g), Pinocembrin (min.1.32–max.39.92 mg/g] and 6 phenolic acid [Caffeic acid (min.1.20–max.7.6 mg/g), p-Coumaric acid (min.1.26–max.4.47 mg/g), trans-Ferulic acid (min.1.28–max.4.92 mg/g), Protocatechuic acid (1.78 mg/g), trans-Cinnamic acid (min.1.05–max.3.83 mg/g), Caffeic Acid Phenethyl Ester (CAPE) (min.1.41–max.30.15 mg/g)] components were detected as mg/g, in different ratios in propolis samples collected from different regions. The feature of this study, so far, is to have the maximum number of samples representing the Turkish propolis, and so is thought to help to national and international propolis standard workings.

## Introduction

Propolis or bee glue is a substance containing a mixture of wax and resin collected by honeybees (*Apis mellifera* L.) from different parts (tree and flower buds, sap flows, mucilage, latex, resin etc.) of plants [1–5]. Honeybees, collect propolis from protective resins of flowers and trees buds with their lower jaws and carry them to the hive in the pollen sacs on their hind legs. They also add substances from their bodies during the resin collection and modeling phase. The collected propolis ensures that the hive is protected from all kinds of diseases and prevents the entrance of insects and animals by closing the small openings in the hive [6, 7].

Propolis, generally consists of 50% balsam, 30% wax, 10% essential oils and 5% pollen. Since the 1950s, scientists have started to isolate important components in propolis with the help of new analytical methods and have shown people that they have many benefits [7]. Propolis and its many compounds show a wide variety of biological and pharmacological activities [8]. It is a supplementary and supportive food that has become popular all over the world in 2000s, thanks to its antimicrobial [9–11], antioxidant [12], anticancer [13], antiulcer [14], antidiabetic [15], anti-inflammatory [16], antigenotoxic [17] and antiviral [18–22] activities. Propolis, which is also used in traditional and complementary medicine, is an important bee product and it is used in “Apitherapy”, which is a treatment method with bee products [23–26]. In particular, it has been determined by many scientists that it is effective on the corona virus in the COVID-19 pandemic in 2020 [18–22]. Therefore, people's interest in propolis has increased more.

The content of propolis varies according to the plant source and when it is collected [27]. In addition, the variety in the content of beeswax affects the chemical composition of the raw propolis [28]. More than 300 different compounds have been detected to date in the propolis [29–31]. The majority of these components are phenolic acids and flavonoids [32].

Plant species in a geographic region determine the amount and type of compounds found in the propolis. In a study in New Zealand, dihydroflavonoids, pinobanksin and pinocembrin accounted for approximately 70% of the flavonoids in the analyzed samples. However, in a similar study conducted in Brazil, Uruguay and China, the dihydroflavonoid in the samples was 10% less than the samples in New Zealand [7]. Moreover, it has been found to vary the amount of flavonoids and phenolic contents in propolis samples collected from different regions of Turkey [33–35]. The most important pharmacological activity elements in propolis are flavones, flavanols and flavanones, which are common names flavonoids, and various phenolics and aromatics [7].

Propolis contains hundreds of different substances with antimicrobial properties, about 80 of which are flavonoids [1, 36–39]. Phenolic compounds in propolis are found in large quantities at about 1 in 3, while flavonoids are only up to 10% (w/w) of the concentrated form of propolis [40, 41]. Among them, pinocembrin and galangin provide antibacterial activity. It also has pinocembrin, fungicidal and local anesthetic properties [36]. Cinnamyl alcohol, cinnamic acid, vanillin, benzyl alcohol, benzoic acid, caffeic acid, coumaric acid and ferulic acid are some phenolics found in propolis [7]. In recent years, studies on propolis have found that pinocembrin, pinobanksin, quercetin, chrysin and galangin flavonoids and caffeic acid and coumaric acid phenolic acids are the most common components in propolis [9, 42–44].

In this study, total flavonoids and total phenolic compounds content and total antioxidant capacity was determined at the 23 propolis samples which were collected different regions of Turkey. In addition, the diversity of phenolic and flavonoid components of these propolis samples was found by HPLC and compared with other studies. Beside that, the similarities and differences of these 23 provinces to each other according to their antioxidant capacities were investigated by multidimensional scaling analysis. On the other hand, there are marketing difficulties for propolis because of the lack of the national or international propolis standard. For this reason, national and international standard studies will progress more easily thanks to studies that reflect the general characteristics of country propolis, such as this study, and this will solve marketing problems.

## Material and methods

### Collecting of propolis samples

**Fig. 1:**
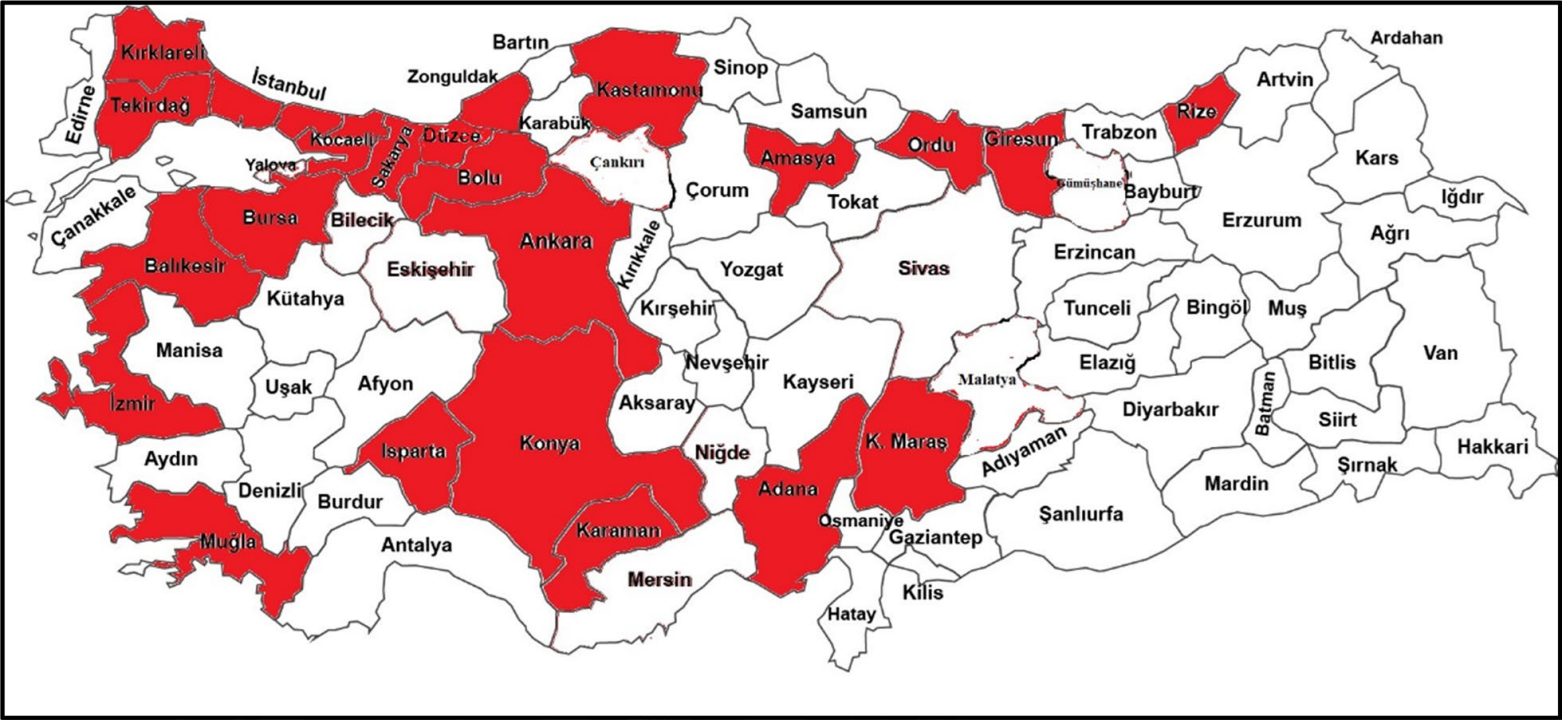
Regions where propolis samples were collected

Propolis samples were collected from 23 different cities in Turkey in 2019. Propolis traps placed in the hives in spring season were harvested end of the summer. Traps were kept in the freezer and were removed from the freezer while preparing propolis extracts (Fig. 1).

### Preparation of extracts from raw propolis

About 30 mL of 70% ethanol solution is added to the powdered 1 g of raw propolis sample, shaken for 24 h at room temperature in shaker. The upper part is filtered through coarse filter paper and transferred to 100 mL flask. The process is repeated by adding 30 mL of 70% ethanol solution to the remaining solid part. The supernatant is added to the flask in which the first extract is collected, completed to 100 mL with 70% ethanol solution [45].

### Total phenolic analysis

Total phenolic content was found by modifying the Meda et al. [46], method. According to this method, the working curve was prepared using varying concentrations (0.25–0.13–0.06–0.03–0.02) of the Gallic acid (GAE) standard (0.5 mg/mL) for calibration. The dilution appropriate for the sample was done with extraction solution and 200 µL of diluted sample was put into the tubes for analysis. For the blank, 200 µL extraction solution was substituted for the sample. For the working curve, 200 µL tubes of varying concentrations of gallic acid were placed in tubes. Then, 1.5 mL of 0.2 N Folin solution was added to the tubes and left for 5 min. The tubes were then vortexed by adding 1.2 mL of NaCO_3_ (7.5%) solution. It was incubated in the dark at room temperature for 90 min. Finally, the  UV-spectrophotometer was read against the curve at a wavelength of 765 nm.

### Total flavonoid analysis

Total flovonoid content was found by modifying the Dewanto et al. [47], method. According to this method, the working curve was prepared using varying concentrations (0.1–0.08–0.05–0.02–0.01) of the catechin (CE) standard (1 mg/mL) for calibration. The dilution appropriate for the sample was done with extraction solution and was placed in tubes from 1 mL of diluted sample for analysis. For the blank, 1 mL of extraction solution was substituted for the sample. For the working curve, 1 mL tubes of varying concentrations of catechin were placed in each tube. The timing is started with the stopwatch and 300 µL of 5% NaNO_2_ (at t = 0 time), 300 µL of 10% AlCl_3_ (at t = 5 time), 2 mL of 1 M NaOH (at t = 6 time) and finally 2.4 mL distilled water was added and vortexed. Without delay, the UV-spectrophotometer was read against the curve at a wavelength of 510 nm.

### CUPRAC antioxidant capacity analysis

CUPRAC antioxidant capacity was detected according to the Apak et al. [48], method. According to this method, the working curve was prepared using varying concentrations (0.5–0.25–0.13–0.06–0.03) of the trolox standard (1 mg/mL) for calibration. Dilution appropriate to the sample was done with extraction solution and 100 µL of diluted sample was put into the tubes for analysis. For the blank, 100 µL extraction solution was substituted for the sample. For the working curve, 100 µL of the varying concentrations of trolox were put into the tubes. Then 1 mL of CuCl_2_, 1 mL of neocuproin, 1 mL of NH_4_CH_3_COO and 1 mL of pure water were added and vortexed, respectively. Incubated for 1 h at room temperature in the dark. Finally, a 450 nm wavelength reading was made on the UV-spectrophotometer.

### HPLC component analysis

Modified Aliyazıcıoglu et al. [33], method was used for propolis HPLC component analysis. Powdered 1 g raw propolis sample is weighed into a 50 mL falcon tube. Add 30 mL of 70% ethanol solution, shake for 24 h on a shaker. After centrifugation, the upper phase is transferred to a 100 mL volumetric flask. The shaking process is repeated once more. The upper phase is added to the volumetric flask where the first extract is collected, and complete to 100 mL with 70% ethanol solution. The solution is filtered through a PVDF syringe filter and transferred to the vial and 20 µL is injected to the HPLC device. VWR Hitachi HLC-UV Detector (UV 280 nm) and Supelcosil LC-18 25 cm × 4.6 mm, 5 µm column is used in the HPLC. Mobile phase A: 99% Ultra pure water: 1% Acetic Acid and Mobile phase B: 100% Methanol is used and flow was 0.9 mL/min, a linear gradient was applied by increasing the B mobile phase from 10 to 90%.

### Statistical analysis

Multidimensional scaling (MDS) is a way to visualize the level of similarity between binary distances between a series of n objects or units. With multi-dimensional scaling analysis, objects are displayed in a k-dimensional (k > p) space based on the distance determined by p variable between n observations or units [49]. In this study, the similarities between the provinces according to the variables phenolic, flavonoid, and CUPRAC parameters were investigated using the multidimensional scaling analysis with Euclidean distance model. The similarity matrix obtained based on the variables in question was used to show the proximity and distance of the provinces to each other. The differences between provinces in relation to flavonoid was researched with One-Way analysis of variance (ANOVA). The Kruskall Wallis test was used for phenolic and CUPRAC because of normality assumptions are not valid. The differences of group means were detected by Duncan and Bonferroni multiple comparison test, parametric and nonparametric analysis, respectively. The IBM SPSS v25 program was used all statistical analysis.

## Results and discussion

### Total flavonoid content

**Fig. 2:**
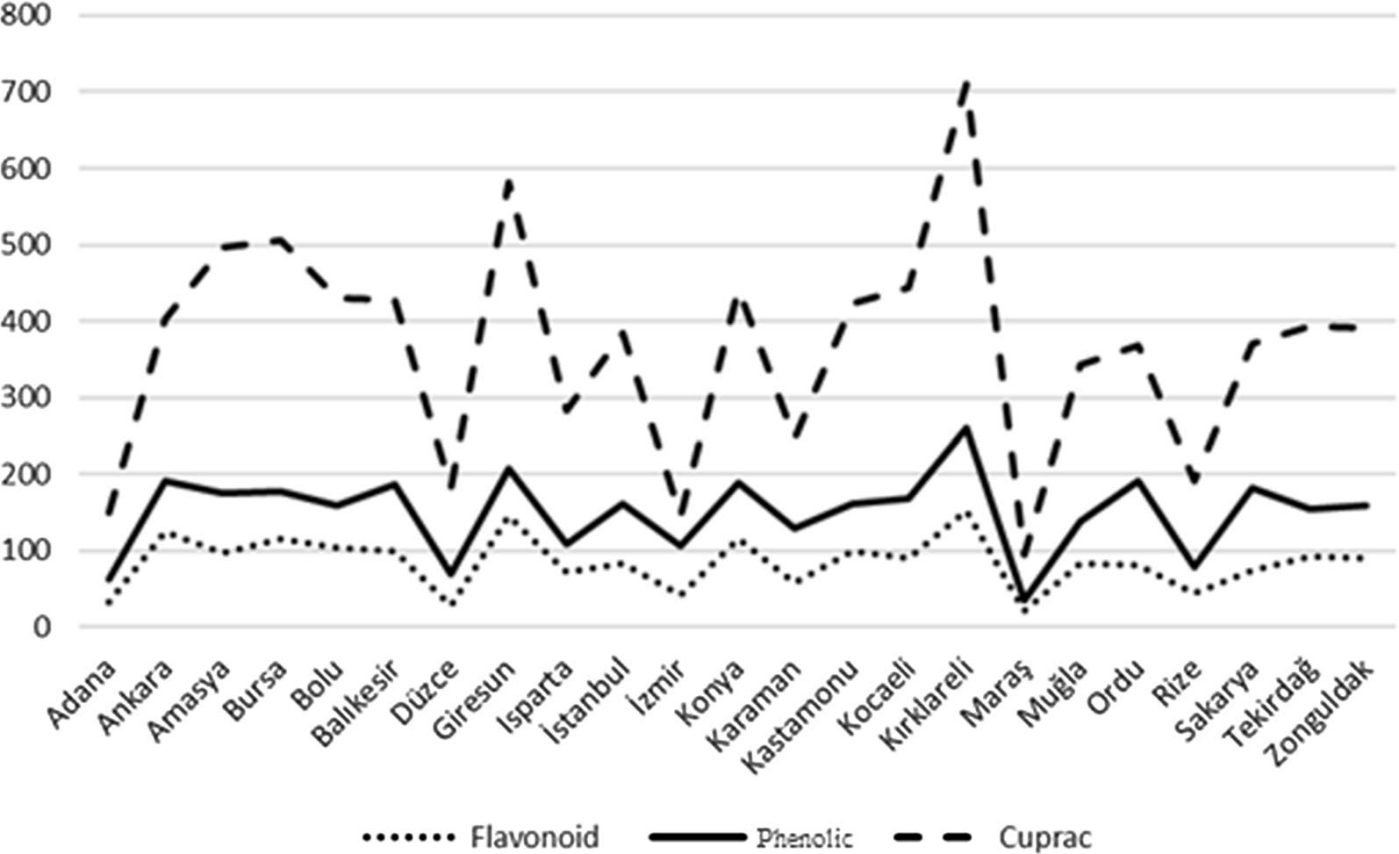
Comparing the flavonoid, phenolic and CUPRAC results according to the provinces

The total flavonoid content in the propolis samples were determined between 21.28 and 152.56 mg CE/g. Kırklareli (152.57), Giresun (146.35), Ankara (125.66) provinces have the highest values, while İzmir (42.1), Düzce (28.35) and Maraş (21.28), provinces have the least values were observed. The average of all provinces were found to be 84.77 mg CE/g value (Table 1, Fig. 2). Many researchers have determined the total flavonoid value in propolis. Some of these researchers and their conclusions are as follows: Zarate et al. [50] in Mexico; 13–379 mg QE/g, Ozdal et al. [51] in Turkey; 522.71 mg QE/g, Narimane et al. [52], in Algeria; 0.57–3.53 mg QE/g, Wang et al. [53] in South Korea; 21–50 mg QE/g and for Brazilia, China and Australia; 33–53 mg QE/g, Socha et al., [54] in Poland; 35.64–62.04 mg QE/g and Bonvehi and Gutierrez [55] in Spain; 72–161 mg QE/g. These results are compatible with our study.

**Table 1:** The ANOVA means and standart errors for flavonoid, and Kruskall Wallis mean ranks for phenolic and cuprac according to provinces

Provinces	N	Flavonoid mg Catechin/g	Phenolic mg Gallic Acid/g		CUPRAC mg TE/g	
		Mean ± sd error	Mean ± std deviation	Mean rank	Mean ± std deviation	Mean rank
Adana	3	32.20 ± 0.21^n^	62.92 ± 0.75	8.00^a^	150.63 ± 0.65	8.00^abc^
Ankara	3	125.66 ± 0.30^c^	191.55 ± 0.79	62.0^abc^	402.65 ± 0.50	41.00^abc^
Amasya	3	96.60 ± 2.54^gh^	175.10 ± 2.70	44.00^abc^	495.87 ± 3.33	59.00^abc^
Bursa	3	115.27 ± 2.09^d^	177.77 ± 3.85	57.33^abc^	506.27 ± 3.81	62.00^abc^
Bolu	3	104.49 ± 0.34^e^	158.58 ± 0.95	53.00^abc^	430.99 ± 1.75	50.00^abc^
Balıkesir	3	99.54 ± 0.31^fg^	186.78 ± 1.55	47.67^abc^	427.86 ± 0.85	47.00^abc^
Düzce	3	28.35 ± 0.74^o^	69.30 ± 3.25	5.00^abc^	182.70 ± 0.84	11.00^abc^
Giresun	3	146.35 ± 0.40^b^	208.20 ± 2.64	65.00^ac^	580.93 ± 3.02	65.00^ac^
Isparta	3	71.40 ± 0.86^k^	108.13 ± 1.98	20.67^abc^	282.30 ± 1.93	20.00^abc^
İstanbul	3	83.52 ± 0.37^j^	161.77 ± 1.86	31.00^abc^	384.07 ± 2.25	32.00^abc^
İzmir	3	42.10 ± 2.14 m	107.20 ± 0.46	12.33 ^abc^	144.93 ± 2.06	5.00^a^
Konya	3	115.50 ± 2.17^d^	187.97 ± 2.18	57.67^abc^	441.53 ± 0.26	54.00^abc^
Karaman	3	57.67 ± 0.72^l^	129.07 ± 2.80	17.00^abc^	245.33 ± 3.51	17.00^abc^
Kastamonu	3	100.18 ± 0.64^f^	160.98 ± 1.56	48.00^abc^	424.00 ± 2.61	44.00^abc^
Kocaeli	3	91.10 ± 0.13^i^	167.21 ± 0.40	37.00^abc^	443.94 ± 0.15	55.00^abc^
Kırklareli	3	152.57 ± 1.47^a^	259.40 ± 1.73	68.00 ^c^	710.43 ± 2.01	68.00^c^
Maraş	3	21.28 ± 1.02^p^	34.53 ± 2.10	2.00^b^	95.35 ± 2.83	2.00^b^
Muğla	3	82.95 ± 0.95 ^j^	139.28 ± 0.67	30.00^abc^	342.56 ± 0.74	23.00^abc^
Ordu	3	80.55 ± 0.48^j^	190.92 ± 0.88	26.00^abc^	368.53 ± 2.50	27.00^abc^
Rize	3	44.04 ± 0.96^m^	78.06 ± 0.86	12.67^abc^	191.13 ± 0.40	14.00^abc^
Sakarya	3	73.93 ± 1.01^k^	182.48 ± 2.69	22.33^abc^	369.57 ± 0.93	28.00^abc^
Tekirdağ	3	93.39 ± 0.53^hi^	155.22 ± 1.01	42.00^abc^	393.62 ± 1.16	38.00^abc^
Zonguldak	3	91.20 ± 0.70^i^	159.82 ± 0.96	36.33^abc^	390.42 ± 1.87	35.00^abc^
p value		< 0.001	< 0.001		< 0.001	

Different letters in the same columns show statistically differences between means (p < 0.05)

### Total phenolic content

The total phenolic content in the propolis samples was found between 34.53 mg GAE/g and 259.4 mg GAE/g. Kırklareli (259.4), Giresun (208.2), Ankara (191.55), Ordu (190.92) provinces have the highest values, while Kahramanmaraş (34.53), Adana (62.92), Düzce (69.3), Rize (78.06) provinces have the lowest values were determined. The average of all provinces were found to be 150.09 mg GAE/g value and statistically Adana, Kırklareli and Maraş were found statistically different from each other (p < 0.05) (Table 1, Fig. 2). There are many studies on total phenolic compound in propolis. Some of them are as follows: Zarate et al. [50] in Mexico; 68–500 mg GAE/g, Ozdal et al. [51] in Turkey; 314.36 mg GAE/g, Narimane et al. [52] in Algeria; 0.81–8.97 mg GAE/g, Wang et al. [53] in South Korea; 49–239 mg GAE/g and for Brazilia, China and Australia; 127–142 mg GAE/g, Socha et al. [54] in Poland; 150.05–197.14 mg GAE/g, Aliyazıcıoglu et al. [33] in Turkey; 115–210 mg GAE/g and Bonvehi and Gutierrez [55] in Spain; 200–340 mgGAE/g. All results are consistent with our study.

### Antioxidant capacity (CUPRAC)

CUPRAC method gives information about reductive capabilities of propolis extracts and based on reduction of Cu^+2^ to Cu^+^ by antioxidants [52]. Table 1 and Fig. 2 show the antioxidant capacity of the propolis samples and antioxidant range was found from 95.35 to 710.43 mg TE/g. Kırklareli (710.43), Giresun (580.93), Bursa (506.26) provinces have the highest values, while Maraş (95.35), Adana (150.63), Düzce (182.7), provinces have the lowest values were determined. The average of all provinces was found to be 365.46 mg TE/g value and İzmir, Kırklareli, and Maraş provinces were found statistically different from each other (p < 0.05) (Table 1, Fig. 2). Researchers found the CUPRAC value in propolis in different countries respectively: Bayram et al. [56] in Turkey; 282.8 mg TE/g, Ozdal et al. [51] in Turkey; 1184.94 mg TE/g, Narimane et al. [52] in Algeria 8 µM TE/g, Daraban et al. [57] in Romania; 12404–35721 µM TE/100 mL. These results are similar to our study results.

### MDS analysis

**Fig. 3:**
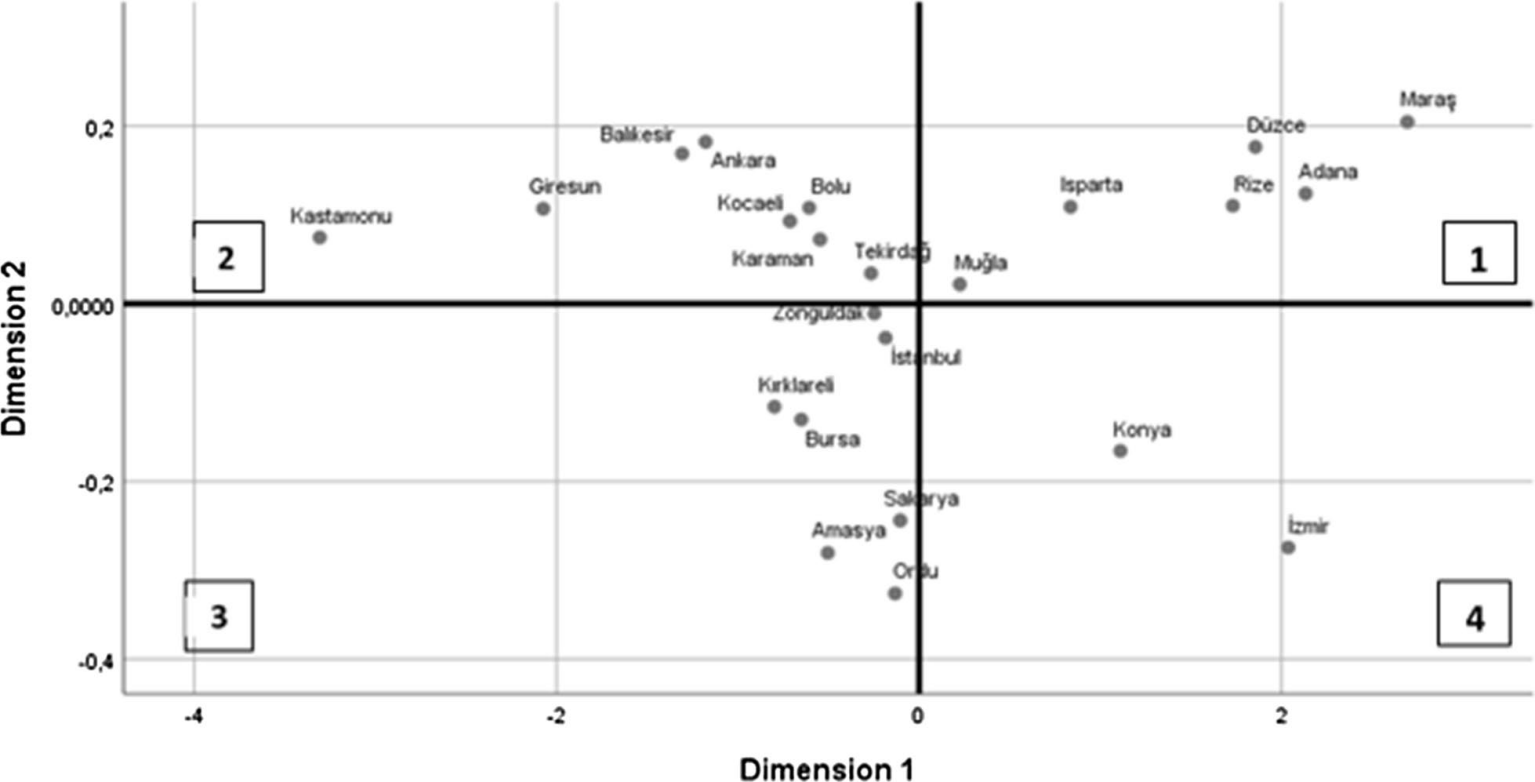
Optimal two dimensional configuration of provinces obtained by MDS

The results of the examination according to the similarities and differences of all provinces according to Flavonoid, Phenolic and CUPRAC antioxidant capacity contents are given in Fig. 3. After several dimensional scaling analysis, two-dimensional (k = 2) scaling was determined the best because of giving the lowest The Kruskal's stress value and higher the coefficient of determination (R^2^), as 0.004 and 0.99, respectively. Therefore, the results were given, and comments were made on two-dimension scaling.

The stimulus coordinates of provinces and configurations of provinces showed that Muğla, Isparta, Düzce, Rize, Adana and Maraş were found similar, Tekirdağ, Karaman, Kocaeli, Bolu, Ankara, Balıkesir, Giresun and Kastamonu were found similar; Zonguldak, İstanbul, Kırklareli, Bursa, Sakarya, Amasya and Ordu were found similar and Konya and İzmir were found similar among each other. Optimal two‐dimensional configuration of provinces based on stimulus coordinates was illustrated in Fig. 3.

### HPLC component analysis

Propolis has many biological and pharmacological activities thanks to its large number of phenolic and flavonoid components [33]. For the HPLC method validation of the study, the repeatability, reproducibility, recovery, linearity, limit of detection limit (LOD) and limit of quantitation (LOQ) validation parameters were examined and presented in Table 2.

**Table 2:** Validation parameters of HPLC

Compound	R^2^	Recovery (%)	LOD (mg/kg)	LOQ (mg/kg)	5 mg/L		20 mg/L		40 mg/L	
					RSR_r_	RSD_R_	RSR_r_	RSD_R_	RSR_r_	RSD_R_
Quercetin	0.9975	87.9	1.14	1.51	0.66	1.89	1.57	1.93	1.81	3.09
Galangin	0.9936	80.0	0.93	1.58	0.72	1.51	0.78	1.67	1.09	1.81
Apigenin	0.9987	104.9	1.13	1.90	0.75	1.01	1.05	1.26	1.16	1.93
Pinocembrin	0.9969	110.2	0.70	1.20	0.78	1.51	1.75	2.27	1.98	2.30
Caffeic acid	0.9983	108.4	0.89	1.45	0.30	1.52	1.22	2.23	1.31	2.93
p-Coumaric acid	0.9980	106.2	0.67	0.93	0.23	1.22	1.32	1.47	1.43	1.68
Trans-ferulic acid	0.9983	98.7	0.56	0.60	0.37	1.12	1.56	1.82	1.78	2.21
Protocatechuic acid	0.9983	108.7	0.51	0.82	0.80	1.18	0.97	1.24	1.07	1.92
Trans-cinnamic acid	0.9984	88.5	0.61	0.66	0.57	1.06	0.91	1.44	1.09	1.76
Caffeic acid Phenethyl ester (CAPE)	0.9983	98.9	0.51	1.18	0.24	1.37	1.45	1.83	1.59	2.37

**Fig. 4:**
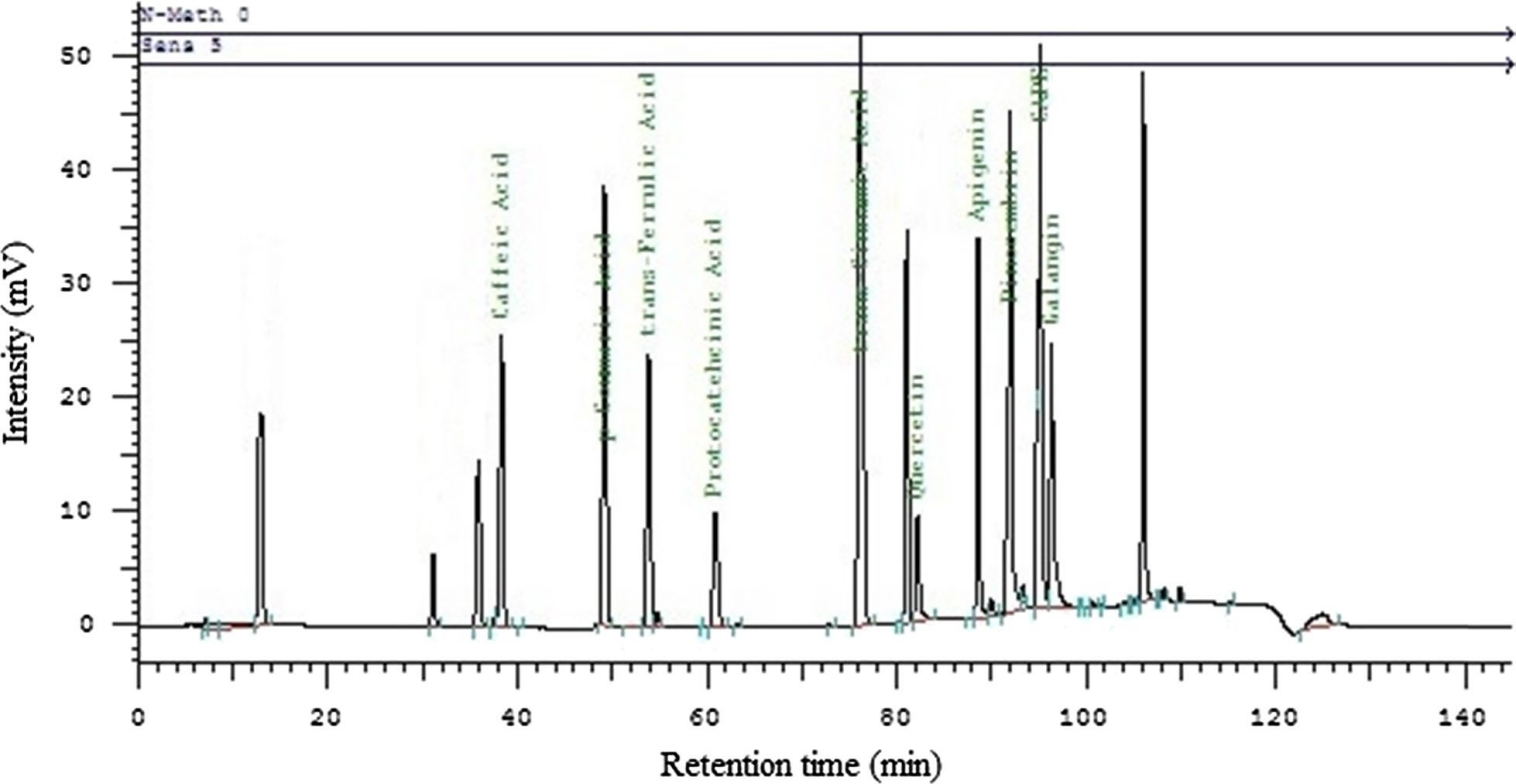
HPLC chromatogram of the components

In this study, 4 flavonoid [quercetin (min.1.12–max.4.14 mg/g), galangin (min.0.72–max.40.79 mg/g), apigenin (min.1.07–max.17.35 mg/g), pinocembrin (min.1.32–max.39.92 mg/g] and 6 phenolic acid [caffeic acid (min.1.20–max.7.6 mg/g), p-coumaric acid (min.1.26–max.4.47 mg/g), trans-ferulic acid (min.1.28–max.4.92 mg/g), protocatechuic acid (1.78 mg/g), trans-cinnamic acid (min.1.05–max.3.83 mg/g), caffeic acid phenethyl ester (CAPE) (min.1.41–max.30.15 mg/g)] components were detected as mg/g, in different ratios in propolis samples collected from different regions of Turkey (Table 3, Fig. 4).

**Table 3:** Propolis component analysis results by HPLC

Provinces	Quercetin (mg/g)	Galangin (mg/g)	Apigenin (mg/g)	Pinocembrin (mg/g)	Caffeic acid (mg/g)	p-Coumaric acid (mg/g)	Trans-ferulic acid (mg/g)	Protocatechuic acid (mg/g)	Trans-cinnamic acid (mg/g)	CAPE (mg/g)
Adana	N.D	1.07 ± 0.39	1.96 ± 0.05	2.14 ± 0.88	4.03 ± 0.2	2.33 ± 0.23	N.D	N.D	2.26 ± 0.5	2.04 ± 0.83
Ankara	N.D	19.24 ± 0.42	1.75 ± 0.04	7.93 ± 0.48	2.01 ± 0.8	1.26 ± 0.03	N.D	N.D	1.20 ± 0.07	19.26 ± 0.66
Amasya	N.D	12.86 ± 0.7	1.81 ± 0.4	6.65 ± 0.45	1.55 ± 0.02	N.D	N.D	N.D	N.D	19.32 ± 0.19
Bursa	N.D	10.87 ± 0.09	N.D	7.25 ± 0.04	4.74 ± 0.07	N.D	N.D	N.D	1.33 ± 0.02	16.08 ± 0.06
Bolu	4.14 ± 0.4	10.42 ± 0.04	1.87 ± 0.3	15.39 ± 1.54	1.32 ± 0.02	2.00 ± 0.05	2.26 ± 0.38	1.78 ± 0.04	1.05 ± 0.02	26.99 ± 0.87
Balıkesir	2.58 ± 0.16	9.64 ± 1.92	1.20 ± 0.05	11.93 ± 3.06	2.83 ± 1.1	N.D	N.D	N.D	1.31 ± 0.07	8.87 ± 0.03
Düzce	1.58 ± 0.28	3.98 ± 0.68	N.D	7.39 ± 0.88	N.D	N.D	1.28 ± 0.07	N.D	N.D	3.65 ± 0.25
Giresun	2.46 ± 0.04	12.52 ± 0.45	N.D	5.42 ± 0.05	4.82 ± 1.4	4.47 ± 0.09	4.92 ± 0.05	N.D	N.D	1.41 ± 0.04
Isparta	N.D	1.58 ± 0.04	1.19 ± 0.07	3.09 ± 0.05	1.20 ± 0.03	N.D	N.D	N.D	N.D	N.D
İstanbul	N.D	7.69 ± 0.4	17.35 ± 0.53	11.07 ± 0.55	N.D	N.D	N.D	N.D	1.80 ± 0.03	3.30 ± 0.38
İzmir	N.D	2.50 ± 0.48	N.D	5.97 ± 0.36	N.D	N.D	N.D	N.D	N.D	1.58 ± 0.05
Konya	N.D	6.12 ± 0.44	N.D	10.41 ± 0.6	2.51 ± 0.6	2.59 ± 0.45	N.D	N.D	1.12 ± 0.04	9.79 ± 0.65
Karaman	N.D	0.97 ± 0.03	N.D	1.32 ± 0.06	N.D	N.D	N.D	N.D	N.D	4.03 ± 0.08
Kastamonu	1.18 ± 0.05	2.77 ± 0.26	1.07 ± 0.06	5.32 ± 0.63	7.6 ± 0.06	2.69 ± 0.09	N.D	N.D	2.13 ± 0.03	19.53 ± 0.04
Kocaeli	1.46 ± 0.28	10.89 ± 5.90	1.19 ± 0.20	29.17 ± 5.08	2.07 ± 0.75	3.47 ± 0.79	2.50 ± 0.43	N.D	2.25 ± 0.85	25.41 ± 4.41
Kırklareli	2.10 ± 0.04	40.79 ± 0.04	4.08 ± 0.06	39.92 ± 0.04	5.77 ± 0.04	3.40 ± 0.26	N.D	N.D	3.47 ± 0.04	30.15 ± 0.04
Maraş	N.D	0.72 ± 0.06	N.D	1.65 ± 0.06	N.D	N.D	N.D	N.D	N.D	3.18 ± 0.18
Muğla	1.14 ± 0.04	6.71 ± 3.73	1.46 ± 0.4	16.32 ± 0.52	1.59 ± 0.07	N.D	N.D	N.D	2.16 ± 1.02	2.13 ± 0.33
Ordu	N.D	4.83 ± 0.32	2.67 ± 0.23	20.22 ± 0.25	1.96 ± 0.7	1.39 ± 0.05	N.D	N.D	N.D	12.54 ± 3.15
Rize	N.D	3.73 ± 0.05	3.77 ± 0.07	17.42 ± 0.07	N.D	N.D	N.D	N.D	2.01 ± 0.05	4.58 ± 0.08
Sakarya	3.89 ± 0.04	16.49 ± 0.03	2.98 ± 0.02	34.82 ± 0.07	2.06 ± 0.04	3.25 ± 0.04	3.26 ± 0.09	N.D	1.15 ± 0.03	15.14 ± 0.05
Tekirdağ	1.12 ± 0.02	11.77 ± 1.56	1.67 ± 0.42	5.88 ± 1.88	5.03 ± 0.02	1.53 ± 0.05	1.32 ± 0.15	N.D	3.83 ± 0.03	21.79 ± 0.35
Zonguldak	N.D	6.95 ± 0.04	N.D	N.D	2.90 ± 0.02	N.D	N.D	N.D	2.55 ± 0.08	N.D

Cunha et al. [58] found caffeic acid, p-coumaric acid and ferulic acid in all of the extracts prepared in their study using different solvents to determine the phenolic content of Brazilian propolis. Also, Choi et al., [59] stated that in many propolis samples obtained from Korea contains caffeic acid (min.1.0–8.7 mg/g), p-coumaric acid (min.1.2–7.1 mg/g), ferulic acid (min.0.5–1.9 mg/g), apigenin (min.0.6–2.4 mg/g), pinocembrin (min.1.5–87.8 mg/g) and galangin (min.4.9–max.26.3 mg/g). These results are in line with our results. On the other hand, Lagouri et al., [60] identified that caffeic acid (min.0.64–max.4.17 mg/g), caffeic acid phenyl ester (min.0.36–max.2.04 mg/g), ferulic acid (min.0.53–max.1.41 mg/g), p-coumaric acid (min.0.83–max.3.00 mg/g), apigenin (min.0.48–max.2.74 mg/g) and galangin (min.1.32–max.8.55) components at the lower amounts according to our study at the Greek propolis samples. Also, Keskin and Kolaylı [34] found caffeic acid (min.0.40––max.7.33 mg/g), ferulic acid (min.0.52–max.9.83 mg/g), coumaric acid (min.0.71–max.4.30 mg/g) phenolic compounds amounts similar to our results at the Turkish propolis samples. On the other hand, Aliyazıcıoglu et al. [33] determined similar results for Turkish propolis samples with our results. They found caffeic acid (1446.8–4658.1 µg/g), p-coumaric acid (381.7–4579.8 µg/g) and ferulic acid (223.3–7126.9 µg/g). Ristivojevic et al. [35] also, found phenolic and flavonoid compounds at the another Turkish propolis study. They revealed caffeic acid (min.3.96–max.34.78 mg/mL), ferulic acid (min.1.00–max.19.42 mg/mL), coumaric acid (min.0.19–max.4.91 mg/mL), protocatechuic acid (min.0.45 mg/mL–max.1.69 mg/mL), trans-cinnamic acid (min.3.00–max.5.28 mg/mL), quercetin (min.1.11–max.4.33 mg/mL), galangin (min.0.96–max.2.70 mg/mL), apigenin (min.0.54–max.1.56 mg/mL), pinocembrin (min.0.94–max.2.81 mg/mL]. Their results were similar to our results. Beside that, Pavlovic et al., [61] determined caffeic acid (min.4.21–max.4.37 mg/g), p-coumaric acid (min.1.40–max.6.97 mg/g), ferulic acid (min.1.64–max.7.41 mg/g), pinocembrin (min.17.90–max.19.06 mg/g) at the Italian propolis samples. Also, these results close to our study results. As a result, in this study, we found caffeic acid, caffeic acid phenethyl ester (CAPE), galangin and pinocembrin as major components for the Turkish propolis. Because these components have been detected in almost all provinces and these components can be used for quality determination and standardization of Turkish propolis. As a similar, Sorucu and Oruc [62] determined pinocembrin, CAPE, caffeic acid highest amounts at the propolis samples from the northwest of Turkey.

As a result, the content of raw propolis varies according to the botanical origin of the region where it is obtained. Turkey, where they grow different plant species, is a country with rich botanical resources. Because, there are three phytogeographical regions in Turkey (Euro & Siberian, Mediterranean, Irano & Turanian) and the plant diversity varies from region to region [63]. So, there are about 12,000 plant species in Turkey and 3000 of them are endemic. About 500 plant species are nectar plants and are preferred by honeybees [64]. Since propolis is in very different phytogeographic regions in the 23 cities studied, the botanic origin varies. For this reason, propolis contents also differ greatly and it is very important to making content analysis for propolis standardization.

In this study, total phenolics, total flavonoids and antioxidant capacity amounts were determined and compared statistically at the propolis samples, which were collected from different regions of Turkey. By illuminating the phenolic and flavonoid components contained in propolis samples, components [caffeic acid, caffeic acid phenethyl ester (CAPE), galangin and pinocembrin] that could be markers for Turkish propolis were determined. Thus, for the basic standardization of Turkish propolis, the range in which the total phenolic substance and flavonoid substance amounts should be and the components it should contain were determined. These compounds can be used in the marketing quality control of Turkish propolis.

## Data Availability

All data analysed during this study are included in this published article.
